# Social Work Responses to Domestic Violence During the COVID-19 Pandemic: Experiences and Perspectives of Professionals at Women’s Shelters in Sweden

**DOI:** 10.1007/s10615-022-00833-3

**Published:** 2022-01-27

**Authors:** Charlotte C. Petersson, Kristofer Hansson

**Affiliations:** Department of Social Work, Malmö University, 205 06 Malmö, Sweden

**Keywords:** Women’s shelters, Domestic violence, COVID-19 pandemic, Shared trauma

## Abstract

This study explores how social work professionals at women’s shelters in Sweden experience, understand, and are responding to domestic violence under the impact of the COVID-19 pandemic. A qualitative longitudinal research design was employed, and multiple semi-structured interviews were conducted with 14 professionals at women’s shelters over a period of one year. The results are presented in three overall themes; (a) professional challenges due to increased needs, (b) professionals’ adjustments to new circumstances, and (c) professionals’ attributions regarding client barriers to help seeking. The results show diverse and changing experiences among the professionals as the pandemic progressed. Clients and professionals have shared the same collective trauma associated with the pandemic, which has affected the professionals’ understanding of and response to domestic violence. The professionals understand both clients and themselves as being more vulnerable and susceptible to risk under these new circumstances. Social work adjustments focused on maintaining contact, reducing risk and prioritizing safety, which had both positive and negative consequences for both clients and professionals. The study concludes that the professionals coped with the uncertainty they experienced during the pandemic by relying on both their previous knowledge and work experience of domestic violence and their experience of sharing trauma with clients.

## Introduction

Times of societal crisis, including economic instability, social unrest, and disaster can be associated with an increased risk for gender violence, a term which refers to a wide range of violations against individuals or groups of individuals based on and embedded in gendered relationships (Merry, 2009). This article concerns the crisis brought on by the SARS‑CoV‑2 pandemic (referred to as COVID‑19) and how it has affected professionals’ work with clients who are exposed to domestic violence—a type of gender violence that encompasses physical, sexual, mental and economic harm against women and children by an intimate partner, parent or caretaker. Since the outbreak of COVID-19, the United Nations (UN) has received reports from various parts of the world concerning an intensification of domestic violence as a result of confined living conditions and tensions related to worries about health, security, and financial circumstances (UN, 2020). Quarantine, lockdowns, school closures, and recommendations to work from home, maintain social distance, and avoid traveling have resulted in some women and children being isolated with a violent and controlling partner or parent in the home. This isolation is believed to make it more difficult for those who are exposed to violence to seek help. The UN refers to this situation as a *shadow pandemic* (UN, 2020). In research and editorials, similar concerns relating to both women and children are highlighted, and the home is described as involving a risk for violence rather than as a safe haven that should provide protection from virus transmission during the pandemic (Bradbury-Jones & Isham, 2020; Krishnadas & Taha, 2020; Hansson & Petersson, 2021). According to the UN (2020), the increase in cases of domestic violence is taking place at the same time as health care services are preoccupied with and focused on providing care to COVID-19 patients. Thus, health care and support services for women and children who are exposed to violence may be disrupted during the pandemic, including the coordination between sectors such as health care, the police, social services, and civil society organizations. Research has noted the importance of having various violence-related services working alongside one another during the pandemic, with these including not only public sector help services but also the voluntary sector (Bradbury-Jones & Isham, 2020). In Sweden, there are more than 200 women’s shelters offering help, protection, and support services to women and children who are exposed to violence. In this article, we explore how professionals at women’s shelters experience, understand, and are responding to domestic violence during the COVID-19 pandemic.

## Domestic Violence and Women’s Shelters in Sweden

Violence against women and children was already a public health issue before the outbreak of COVID-19. Evidence provided by the World Health Organization (WHO) confirms that one in three women in the world have been exposed to physical and/or sexual violence by an intimate partner or to sexual violence by a non-partner at some point in their lives (Garcia-Moreno et al., 2013). Men may also experience violence in the home, but attacks on women and children are often reported as being more severe (Merry, 2009). In Sweden, data confirm that women are exposed to more severe forms of violence and are more likely to be injured than men, which means that women have a greater need for social support and health care services (Frentzel, 2014). The current study concerns the services provided by social work professionals at women’s shelters during the pandemic. Because only a few shelters allow adult men into their organizations, we here focus exclusively on violence against women and children. A recent survey on physical violence against women in Sweden during 2020 shows that offenses reported to the police increased by 3 percent by comparison with the previous year (Swedish National Council for Crime Prevention, 2021). Police reports of physical violence against children did not increase during 2020 compared with the previous year, but reports of sexual violence against children increased by 16 percent (Swedish National Council for Crime Prevention, 2021). During 2020, reports of child maltreatment or neglect submitted to social services^1^ increased by 5 percent compared to the previous year (National Board of Health & Welfare, 2021). Whether crimes and maltreatment reported to the police and social services are correlated with actual levels of violence remains to be investigated further. Data on actual prevalence rates for domestic violence in Sweden during the pandemic are still not available.

In Sweden, the Social Services Act (2001: 453) states that the municipalities have the ultimate responsibility for ensuring that individuals receive the support and help they need, including women and children who are or have been exposed to violence. The Social Welfare Board is responsible for assessing the risk of continued exposure to violence. In cases where children have been subjected to or witnessed violence, such investigations must be carried out without delay (SOSFS, 2021, p. 4). If the assessments show that clients need protection, social services will search for refuge accommodation that is suited to the individual’s circumstances. In this article, the term refuge refers to temporary housing for women and children in acute need of a safe place to stay, away from the perpetrator. In Sweden, several actors provide refuge accommodation to victims of violence, including the private sector, the municipalities, and civil society organizations (women’s shelters) (Ekström, 2016). Social services pay for refuge accommodation in cases in which investigations show that such services are necessary for the client’s safety. The municipalities have different contracts and agreements with the agencies that provide accommodation to abused individuals. Some of the agencies are staffed by social work professionals who can support the clients who are staying in the refuge, while others do not provide such services.

There have been women’s shelters in Sweden since the end of the 1970s and they were developed as a result of the women’s movement, which took the stance that violence against women is a serious patriarchal problem (Merry, 2009). The women’s shelters are organized under two major national organizations (Unizon and Roks), but local women’s shelters are run independently and have their own statutes (Enander et al., 2013). The shelters are staffed by paid professional employees and volunteers. The services offered through women’s shelters are diverse but can be divided into three major areas. Firstly, women’s shelters provide direct help, advice, counseling, and mentoring services to women who are currently in or have recently left an abusive relationship. These social support services are provided both in face-to-face encounters at the organizations and via telephone and online counseling. Secondly, many of the women’s shelters offer temporary refuge accommodation in which social services can place abused women and children in need of protection. Thirdly, women’s shelters conduct preventive work as opinion-makers or by raising awareness and disseminating information about violence to the public. The work of local women’s shelters is financed by grants from municipalities or other authorities, daily fees charged for refuge accommodation, membership fees, and gifts from the public (National Board of Health & Welfare, 2020). In 2017, a total of approximately 2700 women, 2800 children and 30 men were staying in refuges provided by women’s shelters in Sweden (Roks, 2018; Unizon, 2018).

## Social Work and Shared Trauma

Help seeking among women who experience violence in the home is likely to have been affected by the COVID-19 pandemic and the measures introduced by governments around the world to prevent the virus from spreading. Kaukinen (2020) discusses possible help-seeking scenarios in response to domestic violence during the COVID-19 pandemic with a focus on reports to the police. The COVID-19 pandemic and the consequences of family isolation may have increased the incidence and severity of violence, which might in turn result in increased reporting to the police. However, with offenders staying at home, some victims may find it more difficult to find a safe place from which to report incidents of violence, reach out for help, and plan and pack for an escape. Other obstacles may be related to poor financial circumstances due to the pandemic and the fear of retaliation and more violence by the abuser if the victim’s help-seeking efforts are discovered. Kaukinen (2020) also points to the fact that symptoms and illness associated with COVID-19 disease may affect the ability of victims to reach out for assistance.

A review of studies on reported rates of domestic violence during the COVID-19 pandemic has found mixed and inconsistent patterns (Peterman et al., 2020). Rates of domestic violence reported to helpline services have both increased and decreased during the pandemic (see e.g., Krishnadas & Taha, 2020; Southall, 2020; UN, 2020), while other research has found little or no change in calls to domestic violence helplines and to the police (Sorenson et al., 2021). Professionals have reported that shelters and helplines have remained worryingly silent during the pandemic (Bloomberg, 2021) and in a study of domestic violence refuges in Norway, 56 percent of the participants reported a decline in the number of contacts and requests for support from clients during the initial stage of the pandemic (Øverlien, 2020).

While the Swedish government did not enforce a total lockdown of society as a whole, periods of strict restrictions such as school closures and recommendations to work at home, maintain social distance, avoid meeting new contacts, and avoid public transport and crowded settings have still impacted the lives of Swedish citizens and resulted in home isolation. Such restrictions affect not only clients who are living with violence, but also social work professionals. Women and children who are experiencing the trauma of violence during the pandemic are doing this in a context that can also be characterized as traumatic. Professionals are practicing social work, i.e., providing help, support and counseling to their clients, in this trauma-saturated context brought on by the pandemic. In this sense, clients and professionals are experiencing the same collective trauma of the pandemic while dealing with the individual trauma of violence.

Baum (2010) has drawn attention to the concept of “shared trauma” or “shared reality” and how it has been employed by scholars to discuss the impact of collective disasters, such as armed conflict, terror attacks, or natural disasters, on practitioners’ personal and professional experiences. The shared trauma concept emerged, in particular, as scholars employed the term to discuss the terror attacks of September 11 (e.g., Altman & Davies, 2002; Saakvite, 2002; Tosone & Bialkin, 2003). The shared trauma concept has also been employed when referring to professionals’ experiences of practicing social work during the COVID-19 pandemic (Tosone, 2021). Shared trauma does not mean that the clients’ and professionals’ experiences and responses are equivalent. According to Tosone (2012, p. 625), the term captures “the affective, behavioral, cognitive, spiritual, and multimodal responses that mental health professionals experience as a result of primary and secondary exposure to the same collective trauma as their clients.” This dual exposure to trauma creates a situation in which social work professionals may be affected by the blurring of professional and personal boundaries in relation to their clients, increased self-disclosure in relation to clients, and the development of posttraumatic stress (Baum, 2010; Saakvite, 2002; Tosone, 2021). Bell and Robinson (2013) have shown that counsellors are at greater risk for re-traumatization when they share collective trauma with clients. They also found that professionals’ objectivity, empathy and professional engagement may decrease. On the other hand, research by Jenkins and Phillips (2008) concerning intimate partner violence during and following Hurricane Katrina has highlighted the creative role of professionals in relation to women’s help seeking. Their study shows how advocates found new ways to respond to dramatic changes in order to help and meet the unique needs of women who experience violence. In fact, positive experiences can emerge from professionals’ exposure to trauma and their work with clients in this situation, which has been defined as “shared resilience” (Nuttman-Shwartz, 2014). The interaction between professionals and clients who share the same collective trauma can result in mutual aid and positive attitudes and emotions (Nuttman-Shwartz, 2014; Tosone, 2021).

Understanding social work responses to domestic violence under the impact of the COVID-19 pandemic requires data from diverse sources that cover the period of the crisis, including not only statistical reports on prevalence rates for violence, self-reported victimization, and data from social services, but also data describing the experiences of professionals working at civil society organizations. Thus, in this article our aim is to explore how professionals at women’s shelters experienced, understood, and responded to their clients’ help-seeking during a period in which they were sharing a collective trauma with their clients. Focusing on what the social work professionals did in order to keep going under such circumstances, how they worked to provide and maintain their services to women and children who are abused, and how they coped with various challenges and difficulties brought on by the pandemic, provides important insights into the experiences of a group whose voices as care providers are seldom heard.

## Method

This research has employed a qualitative longitudinal research design. It is based on multiple interviews conducted with professionals at women’s shelters over a period of one year, starting in March 2020 at the time of the outbreak of COVID-19 in Sweden. A longitudinal qualitative research design includes the time aspect in the research process and allows for the analysis of change (Thomson et al., 2003). Longitudinal qualitative research captures both “*time and texture*—or the interplay of the temporal and cultural dimensions of social life” (Neale & Flowerdew, 2003, p. 189). By including the aspect of time, we can grasp how individuals understand and relate to crises or rapid social change, the mechanisms individuals or organizations use to generate, cope with, and manage change, and also how change shapes and influences individual lives (Neale & Flowerdew, 2003). This approach has been particularly important in relation to the conduct of our research during the pandemic, which has been characterized by successive waves of infection and concomitant fluctuations in restrictions that may affect the work of the participants and their experiences.

### Participants

The study’s findings are based on telephone interviews with professionals working at 14 women’s shelters—one professional from each shelter. The women’s shelters that participated in the study form a part of civil society. They vary in size and are geographically spread across Sweden. Purposive sampling (Hays & Singh, 2012) was used to recruit participants. The sample selection was based on women’s shelters that work actively with the provision of direct social support services to women and their children that experience domestic violence. Thus, all of the women’s shelters in the sample provided peer support and mentoring services to abused clients by means of direct meetings at their organizations or via telephone helplines and digital counseling. Of the 14 women’s shelters that participated in the study, 13 also provided refuge accommodation.

### Data Collection and Analysis

The sample was recruited by contacting the women’s shelters with an e-mail in which the study and its objectives were presented. Thereafter, the managers of these organizations were asked if they would be willing to participate in the study. The telephone interviews were carried out in two phases. The first phase of interviews was conducted by the second author between March and June 2020 and involved two interviews each with five participants.^2^ During this initial phase, the interviews were kept short, lasting between 10 and 20 min, in order to minimize the workload involved in research participation during a time of crisis. In September 2020, the project was expanded, and additional women’s shelters were included in the research. In this second phase of interviews, between September 2020 and March 2021, the first author was responsible both for interviewing new participants and conducting follow-up interviews from the first phase. Thus, a total of 14 professionals were interviewed in both September/October 2020 and in February/March 2021. The interviews in this phase were longer, approximately 30 min, because the participants were now able to provide deeper and more reflective answers. The interviews were semi-structured and based on an interview guide, which meant that the same questions could be posed to all participants. The same questions were asked at all of the interviews in order to capture possible changes. The main questions included: How has your work been affected by events related to COVID-19? What decisions have been made to address changes and challenges related to COVID-19? Have encounters with, or support services for, clients been affected by the events or decisions that you have mentioned? Have you experienced any changes related to the help-seeking behavior of clients which may be associated with COVID-19? Additional questions were asked, and probing was employed to help respondents develop their answers.

The interviews were digitally recorded and transcribed verbatim in Swedish. The transcripts were compared with the digitally recorded interviews to ensure accuracy. The first author was responsible for carrying out the analysis, including organizing the data by reading the transcripts several times in search for recurring patterns in the conversations. The first author identified themes and sub-themes, which were presented to and re-organized in discussions with the second author. The data analysis was guided by a *trajectory analysis*, as recommended by Grossoehme and Lipstein (2016) for longitudinal studies analyzing individuals’ experiences over time. In our case, this started with a thematic analysis as described by Braun and Clarke (2006). Themes or patterns within the data were developed on the basis of similarities in the participants’ narratives rather than by trying to fit the conversations into a pre-existing coding scheme. In other words, the coding was inductive (Hayes, 2000). Three overall themes, including related sub-themes, were developed to meet the aims of the study. Once the thematic analysis had been completed, the longitudinal analysis began, which focused on how the data included in the themes emerged, changed, or did not change over time (Grossoehme & Lipstein, 2016). Both authors were involved in the interpretations of the quotations presented in this article. The quotations were translated into English subsequent to the conclusion of the analysis.

### Ethics

Research involving human participants must be conducted in accordance with ethical standards to avoid harms and wrongs. In this research project, informed consent was provided by all participants prior to their participation in the research project. Participants have been fully anonymized, and pseudonyms are used. The anonymous data have been stored and archived securely by the researchers in accordance with rules specified by Malmö University and the Swedish Ethical Review Authority. Ethical approval was provided by the Swedish Ethical Review Authority in the form of a statement, which concluded that no risk of harming participants could be foreseen, and the research did not involve any personally sensitive data (Dnr 2020-01533). The participants in the research project are professionals, and the research questions concerned the professionals’ work experiences. No harm or distress was noted or reported during or after the interviews.

## Results and Discussion

The findings from the study have resulted in the development of three major themes, and related sub-themes, namely (a) professional challenges due to increased needs, (b) professionals’ adjustments to new circumstances, and (c) professionals’ attributions regarding client barriers to help seeking.

### Professional Challenges Due to Increased Needs

#### Experiencing and Understanding Increased Requests for Social Support Services

During the spring of 2020, in relation to the start of the pandemic in Sweden, the five professionals that participated in this initial phase of data collection expressed uncertainty about how the pandemic and related social interventions would affect women’s needs for support (Hansson & Petersson, 2021). The interviews that were conducted during the second phase produced clearer results. Of the 14 participating professionals, 8 reported an increase in the number of individuals who had contacted them directly in search of counseling and peer support and mentoring services. Olivia said:

More women call us and are worried. If you have lost your job, there will be further anxiety, further complexity due to the economic situation. There has been a greater complexity due to such things in general in the conversations. People are more worried, more exhausted, everyone has been working from home, you are exhausted in a completely different way in the relationships. (Olivia)

Olivia emphasized the impact of new stressors on clients who are experiencing violence in the home. She understood the increased number of women who contacted them by listening to clients’ narratives of confined living conditions and home isolation. Emma, who also works at a shelter at which they have experienced an increase in requests for social support services, expressed her ideas about new stressors among clients in the following way:

There are both more new women who call us and women we already know, who need more support, conversations, and meetings. We can see that they have problems getting the formal support that they need. There are problems with custody investigations, finances, and the right to compensation from social services. It’s a combination of the pandemic, it feels like the country’s social services do not have sufficient muscle right now. (Emma)

Both quotations above indicate that women who are abused are always under stress, but the professionals experienced that the new situation associated with COVID-19 was placing additional pressure on these women. Emma understood her experience of the increase in requests for help from clients by relating it to the way public support services are being challenged—which has been referred to as a shadow pandemic by the UN (2020). Her observations can be understood as an assessment or attribution, even if they are not a direct experience. Jessica shared similar ideas:

This summer, our services where abused women and children can come or contact us directly have increased by 70 percent. There have been people who just show up here, or people who call us in panic because they have contacted social services, but they do not have enough staff, so the women have not been able to go there. (Jessica)

Jessica also referred to the challenges faced by public support services as an explanation for the increase in requests for help from clients at their shelter. As a result, staff experienced periods during the pandemic when the situation was chaotic. Other participants emphasized that it was not the increase in requests for social support services and the overwhelming feelings this may result in that was problematic. Rather, it was the new context of restrictions that made their increased workload particularly challenging. Jennifer explained:

Staff have been ill, and then you notice how vulnerable the organization is. We have no temporary staff we can call. So it has been a tough spring for us with a lot of extra work, in particular when we have had women and children staying in our refuge. Employees have had to stay at home to take care of their children or themselves when they have been ill, or if they belong to a risk group. We have had to help one another and postpone many things, prioritized as well, “What is most important in our organization?” It is our clients, the women and children who seek support and who are staying with us, they are most important. (Jennifer)

While the other quotations above draw attention to the way in which professionals understand their clients as being more vulnerable during the pandemic, resulting in an increased need for help, support and counseling, Jennifer’s narrative draws attention to the vulnerability of staff. Being exposed to the same communal trauma (e.g., Baum, 2010; Saakvite, 2002; Tosone, 2021), both clients and professionals were now vulnerable. The professionals had to take care of and provide safety to not only their clients but also themselves, resulting in an increased workload. Jennifer expressed that they prioritized their clients at times when they themselves were also stressed and affected by the pandemic.

#### Experiencing Vicarious Trauma as Case-Severity and Complexity Increase

Several participants reported that they experienced the violence to which women and children were being exposed during the pandemic as more severe. This was particularly evident in the interviews conducted during the second phase of data collection. These experiences were based on what help-seeking women and children had told participants in conversations over the phone, via the internet or in face-to-face encounters. Sarah described the following:

In our conversations with women, we hear that both women and children are exposed to serious physical and sexual violence, and there are also incidents of incest from the children’s father. Women describe that the violence has escalated very quickly. It takes less time before the violence happens again because you are living close together now in a completely different way. Our experience of the impact of the pandemic is that the women wait until the last minute before leaving, that the violence has really got to the level where we are talking about very serious physical violence with the risk of fatal outcomes. (Sarah)

Sarah experienced a change in the clients’ narratives of violence. She described how professionals experienced their clients as being more vulnerable and susceptible to severe forms violence during the pandemic. This seemed to evoke a sense of helplessness among the professionals to reach clients who they perceived to be in danger. Jennifer explained her experiences as follows: “We have experienced more calls and enquiries than previously, and these have concerned severe forms of violence, more severe now than previously. The cases are more complex and have required a lot on our part.” According to Jennifer, there was a complexity of issues surrounding the women, which affected their work both professionally and personally. Each case required more time and engagement on the professionals’ part. Jessica revealed what it is like to support the women and children who seek help in such severe circumstances:

They call us in panic because the person who is abusing them is working from home and they cannot get out of there. We have had to alert the police. There have been some children who have called and said that their father beats their mother and that they do not know what to do. We had a very difficult conversation with a child whom we estimated to be less than 10 years old, who shouted down the phone “help me, help me, he will kill my mother” and then the line went dead. The staff felt so bad that we had to call in our supervisor [a psychologist]. It has been a completely different challenge for us during the pandemic. (Jessica)

Professionals expressed that the increased vulnerability, the increased severity of violence and the increased complexity surrounding each case put the staff at women’s shelters in a more demanding and difficult position, not least with regard to knowing how to respond to situations in which there may be a risk of a fatal outcome. Jessica explained how staff experienced fear and anxiety on behalf of the clients they were working with. They were doing this at a time when they themselves were susceptible to vulnerability, i.e., while being exposed to the same collective trauma as their clients. In this sense, the professional social workers were experiencing primary trauma as members of a society affected by COVID-19. They also experienced secondary trauma as professionals providing services to clients who were exposed to violence under these circumstances (Baum, 2010; Saakvite, 2002; Tosone & Bialkin, 2003). In this sense, the professionals experienced a double exposure to trauma (Nuttman-Shwartz, 2014). The professionals related to and understood their clients’ vulnerability on the basis of the collective trauma they were sharing with their clients. In this situation of uncertainty, the professionals expressed their own fear about whether they still had the capacity to intervene effectively in potentially fatal situations.

### Professionals’ Adjustments to New Circumstances

#### Creativity to Facilitate and Encourage Help Seeking

Many civil society organizations in Sweden received government funding at an early stage of the pandemic to adjust their services and meet the needs of vulnerable populations (Government Offices of Sweden, 2020). Thus, there was a general expectation in society that the crisis would produce some form of change in relation to vulnerable populations, including women and children who are abused. Early on, in the first phase of data collection, participants described how they had to adapt and make adjustments:

We expanded our digital chat function straight after the WHO announced the pandemic, in early April. We have had a chat function for adolescents for many years but now we started one for adult women as well, with financial support from the government. The funding was channeled through our national organization. (Sarah)

What we have done is that we have started—we have not had so many digital tools before, so now we have started up both a Facebook and an Instagram account. We have not started a digital chat function yet, but it is in progress. (Amelia)

Sarah and Amelia explained how they had improved their accessibility by opening new digital chat functions or by opening new social media accounts. Other participants added that they had also increased their opening hours so that clients could contact them more easily. They also increased their visibility in the media via advertisements and by publishing informative articles. In an interview conducted in the second phase of data collection, Jessica said: “The pressure of people seeking contact at our center is still very high and there are many adolescents in particular who are utilizing our digital chat function.” Mary had noticed similar patterns:

There are more women seeking our support now during the pandemic and it is young people in particular who are contacting us. We opened up to social media this fall, and this may have led to younger women coming to us for advice and support, women who have not found us before. (Mary)

By responding quickly to the new circumstances produced by COVID-19, as has previously been described by Jenkins and Phillips (2008) with regard to Hurricane Katrina, the professionals at women’s shelters adapted their work to facilitate and encourage help seeking among clients (Turner, 2021). As a result, some women’s shelters have experienced an increase in requests for help from certain groups of clients, and not least young women.

While the adoption and usage of more digital tools improved the ability to establish and maintain contact with clients, participants also explained that this had changed their way of interacting with clients. Olivia, who provided digital counseling using the video platform Zoom, experienced the following:

All of a sudden, I was in the home of the client [through Zoom]. I experienced the conversations to have another content and to be less professional, less focused than under ordinary circumstances. Cats, children, noises, interrupting phone calls, bad wi-fi connections, and so on, disturbed our conversations. […] Clients were now asking me questions about my life and how I was managing during the pandemic as if we were friends—this had never happened before. (Olivia)

Oliva emphasized how the space in which the professional counseling was taking place changed dramatically with the usage of digital forms of counseling. She also addressed how her relationship with the clients had changed and how their intimate world became a part of the professional conversation (Hansson & Bjarnason, 2018). The narrative provides an example of how the professional and personal boundaries between clients and professionals are blurred as practitioners share collective trauma with their clients (Baum, 2010; Saakvite, 2002; Tosone, 2021).

#### Managing Risk of Infection and Risk of Violence

From an early stage of the pandemic, the professionals had to manage their sense of the risk of infection and of spreading the disease, both in relation to themselves and their clients. Both clients and professionals share the same communal trauma, which means that they are exposed to the same risk of infection and must follow the same social restrictions on interaction. To avoid becoming infected, Sarah described how they had made adjustments at their shelter:

Initially, we had to create new routines, “What happens if we suspect that a staff member or a volunteer is ill?” Regarding the support we provide directly to the clients at the shelter, we wanted to continue with the counseling but encouraged conversations by phone instead of having women and children coming here physically, which was also what many clients said themselves, “Can we take it over the phone.” In this sense, we have minimized direct contact with clients, but we have also been able to ensure that our staff are healthy, and that there are staff who can work here. (Sarah).

Maintaining contact was central for both clients and professionals during times of isolation and distance, and Sarah described how clients and professionals shared a mutual understanding when developing solutions to the problem of maintaining distance. Here we see clients and professionals helping each other to prevent the risk of infection, which represents a positive adjustment and a positive adaptation to the new situation (Nuttman-Shwartz, 2014; Tosone, 2021). However, when clients needed to stay at the refuge, maintaining distance became difficult, particularly since many refuges are based on the idea of cohousing, which means that the residents have common spaces and share certain facilities. Sophia revealed: “We have fewer women staying at our refuge partly because we have chosen to limit the number due to the risk of spreading the infection.” As has been demonstrated elsewhere, the risk of becoming infected also hindered many of the social and pedagogical group activities arranged by the professionals at women’s shelters to strengthen and empower women who are abused and to activate their children (Hansson & Petersson, 2021).

Another form of risk that professionals addressed was the clients’ risk of being exposed to more violence if the perpetrator were to find out that the woman was in contact with the shelter. Some participants highlighted how they had to change their routines for contacting clients during the pandemic. Mia described this as follows: “We have had a couple of conversations where we had to be extra careful when calling or texting the women, to not write or say that we are from the women’s shelter and so on.” Making arrangements to avoid revealing the client’s contacts with professionals is more important in times when the perpetrator may have more control over the woman. The professionals understood the risk for violence in the home as being more severe during the pandemic and they addressed this risk from the perspective of a worst-case scenario.

### Professionals’ Attributions Regarding Client Barriers to Help Seeking

#### Experiencing and Understanding the Lower Uptake of Services

Not all women’s shelters in the study experienced an increase in the number of help-seeking clients. In fact, the results vary in this regard. Jennifer said:

The number of individuals who call us directly has decreased even though we have increased our availability. We have increased our evening opening hours, the helpline and the digital chat. We have increased the number of volunteers working, but still the number of individuals contacting us has decreased. (Jennifer)

The shelter at which Jennifer works responded to possible outcomes and expectations that the pandemic might produce in the form of an increased need for help among women who are abused. They adjusted their work to these possible outcomes by increasing their availability, but instead fewer women contacted them. The lower uptake of services among the participating shelters concerned their refuges in particular. Of the 13 women’s shelters in the study that offered refuge accommodation, 10 reported that they had fewer women and children staying with them in 2020 than in the previous year. These results are evident in the interviews from both phases of data collection. Some of the professionals reported significant changes, including refuges that had been empty for several months. Emma said: “Occupancy is at least 60 percent lower now and the length of the stay is much shorter.” A few shelters explained the reduction by referring to discourses regarding the general or seasonal fluctuations that tend to influence women’s help seeking under ordinary circumstances (Koutaniemi & Einiö, 2019). Others argued that the changes had been dramatic and can clearly be linked to the pandemic. Susan pointed out that: “There are always differences up and down, and we always compare what it looks like during certain periods or seasons, but from the beginning of the pandemic we experienced that it became very quiet here—the phone went dead.” Sophia explained: “There are very few inquiries from municipalities [concerning refuge accommodation] and it is partly related to the pandemic. It is also linked to the negotiations that the municipalities have had with private refuges.” What Sophia means here is that the low level of requests for refuge accommodation cannot be explained only by reference to the impact of the pandemic but must be understood in the context of a broader perspective that also includes the financial circumstances of the municipalities. Depending on the individual circumstances of the client, social services may choose to place victimized women and children in private refuges, hostels and hotels, where costs may be lower compared to women’s shelters, many of which are staffed by paid social workers.

The absence of women’s narratives about violence in their everyday lives made it difficult for the professionals to know why clients were not asking for help any longer. Mia explained: “We think about what it looks like in people’s homes, what it is like for these women and children, but we do not know for sure. We can only speculate”. The absence of women’s narratives created an uncertainty among the professionals—an uncertainty that grew stronger with time. The lack of contact with clients also created a sense of helplessness among the professionals. Amelia said in an interview conducted during the second phase:

When it comes to the refuge, there is still low occupancy, unfortunately, and the reason I say unfortunately is because we know that violence still exists, and you wonder where the victims are. The violence has not stopped, but rather increased, we think. Whether it’s because women are not shouting for help or if it’s because the municipalities are saving money, we do not know. We interpret the silence as a result of the constant presence of a controlling man. (Amelia)

A controlling partner who prevents help seeking has long been described as a barrier for women to reach out for help. This difficulty was expected to increase as a result of the consequences of family isolation during the pandemic (Kaukinen, 2020). Amelia understood the absence of clients by referring to these circumstances. However, this is an attribution, based on Amelia’s previous knowledge of and work experience with domestic violence.

#### Attributions Regarding How Clients Perceive Uncertainty

The COVID-19 restrictions have produced a range of uncertainties for households and individuals across Sweden. The home isolation directive and the economic implications of the COVID-19 restrictions may have limited not only women’s ability to seek help but also their motivation and readiness to leave a violent partner. This sub-theme emerged only in the second phase of data collection, i.e., when the pandemic had been going on for some time. Sarah linked the low level of requests for refuge to client barriers in the following narrative:

When we talked to the social services, it appears that they have had great difficulty in motivating especially single women to accept a placement at a refuge. They have had women in need of protection, but the women have been ambivalent. In the end, they have chosen to go back to the relationship. (Sarah)

Sarah was referring to what she had heard in conversations with the social services. The quotation demonstrates how professionals perceived an uncertainty among clients during the pandemic, and that this uncertainty may have affected clients’ decisions about the use of possible help services. Camilla also drew on similar experiences when explaining the following:

I think it is very difficult with motivation to be honest, because everything is so uncertain right now. Many women say that: “I did it when the children were at school so I could pick them up, and we could escape together” but now everyone is home. I think it is much harder to leave now. It is so difficult under ordinary circumstances, and now you also have the risk of infection. I mean, do you really want to end up in a refuge now? (Camilla)

Camilla was referring to her previous knowledge of and work experience with domestic violence, together with how she experienced the risk of both infection and violence during the COVID-19 pandemic. She understood clients’ barriers to help seeking by imagining additional difficulties faced by clients. Again, the quotations in this section reveal that professionals understand their clients as being doubly exposed to trauma as a result of COVID-19. They are exposed to both the trauma of violence in the home and to the collective trauma of the pandemic in society. Both Sarah’s and Camilla’s attributions provided them with explanations for the lower uptake of services at their shelters and helped them to understand and cope with the uncertainty and helplessness they experienced in relation to their clients, themselves and their work.

## Conclusion

In this article, our aim has been to explore how professionals at women’s shelters in Sweden experience, understand and are responding to domestic violence under the impact of COVID-19. From the perspective of these professionals, the pandemic and the control measures imposed by the Swedish government have significantly affected their clients’ requests for help and support. Participants identified an increased level of requests for social support services, an increased complexity in the needs of abused women, and an increase in the severity and escalation of violence in the home. On the other hand, professionals also described how they perceived home isolation to have inhibited the ability of clients to seek help and leave the perpetrator. In particular, the professionals noted a decrease in the number of requests for refuge. These may seem to constitute diverse and inconsistent patterns, but similar reports have in fact been described in other studies (Peterman et al., 2020). The professionals explained the changes in women’s help seeking by relating them to their notions and experiences of clients as being more vulnerable during the pandemic. Their clients were exposed to the trauma of violence in the home but also the communal-level trauma resulting from the pandemic. The professionals share this communal trauma with their clients as they provide them with counseling and support. The professionals understand their clients’ vulnerability on the basis of their own dual exposure to primary and secondary trauma (Baum, 2010; Saakvite, 2002; Tosone, 2012, 2021).

Providing social work in response to domestic violence under the impact of COVID-19 had significant implications for practice. Professionals at women’s shelters responded quickly to the new circumstances, even though there was no firm knowledge about the actual impact of the pandemic on the everyday lives of abused women. In order to both encourage and facilitate client help seeking, women’s shelters made adjustments to increase their visibility and accessibility. They provided more social support services and altered the ways in which they provided their services. In order to maintain contact, reduce risk (both for violence and infection), and prioritize safety, multiple adaptations and adjustments were made. Digital counseling was a positive adjustment for both clients and professionals, based on empathic, mutual, and bidirectional aid (Kingstone & Dikomitis, 2021; Nuttman-Shwartz, 2014; Turner, 2021). On the other hand, one participant pointed out that digital counseling also resulted in a blurring of the boundaries between the professional and the client (Hansson & Bjarnason, 2018; Tosone, 2021; Tosone & Bialkin, 2003). In order to maintain the provision of social support services, the professionals had to rely on their previous knowledge and understanding of violence, and of how violence manifests itself in the lives of abused women and children under ordinary circumstances. The study’s findings also show how the professionals turned to their own understanding and experience of the pandemic, and the collective trauma they shared with clients. Attributions provided them with explanations for the lower levels of service uptake at their shelters, and helped them to understand and cope with the uncertainty, helplessness and the risks they experienced in relation to their clients, themselves, and their work.

Conducting research in times of societal crisis involves methodological limitations. The interviews for this study were exclusively conducted by phone. Face-to-face encounters with professionals were not possible and fieldwork in the form of participant observations at the shelters were excluded due to the risk of infection. Different research methods would have generated different data and possibly also “thicker descriptions” (Geertz, 1973). However, by conducting multiple telephone interviews over a long period of time, we found that interviewees were nonetheless able to provide reflective and expansive responses to our questions. The interviews changed over time, including their content. In the first phase, the interviews were deliberately kept short to reduce the workload of the participants, who at this initial stage of the pandemic had to manage dramatic changes to their organizations and to their social work provision. In the first interviews, professionals tended to focus on the practical issues they were facing. The interviews in the second phase were longer, more reflective, and mirrored the implications of the changes that had been made. By employing a trajectory analysis (Grossoehme & Lipstein, 2016), we have examined how the data emerged over time, and how these data changed or did not change. Further research that continues to explore the social work response to domestic violence in a post-pandemic environment will be needed in order to understand and analyze the long-term implications of the pandemic. Once the crisis has passed, it may be easier to shed light and reflect on the positive consequences of providing social work while sharing trauma with clients, such as identifying various aspects of professional growth or resilience.

## Footnotes

1 In Sweden, employees at certain authorities or units that come into contact with children or their parents have a legal responsibility to report child maltreatment or neglect to the social services. The public can also report child maltreatment or neglect to the social services but are not legally obliged to do so.

2 Research results based on data from the initial three months of the project have been published in Swedish and focus on how women’s shelters came to mobilize in order to conduct social work while at the same time maintaining social distance. The study explores how the relationship between abused women and the community can be understood during the COVID-19 period by applying a theoretical model of immunitary life (Hansson & Petersson, 2021).
